# Neuroinflammation and neurodegeneration in diabetic retinopathy

**DOI:** 10.3389/fnagi.2022.937999

**Published:** 2022-08-16

**Authors:** Lorenzo Bianco, Alessandro Arrigo, Emanuela Aragona, Alessio Antropoli, Alessandro Berni, Andrea Saladino, Maurizio Battaglia Parodi, Francesco Bandello

**Affiliations:** Istituto di Ricovero e Cura a Carattere Scientifico (IRCCS) San Raffaele Scientific Institute, Vita-Salute San Raffaele University, Milan, Italy

**Keywords:** diabetic retinopathy, neuroinflammation, diabetic retinal neuropathy, neurodegeneration, diabetes

## Abstract

Diabetic retinopathy (DR) is the most common complication of diabetes and has been historically regarded as a microangiopathic disease. Now, the paradigm is shifting toward a more comprehensive view of diabetic retinal disease (DRD) as a tissue-specific neurovascular complication, in which persistently high glycemia causes not only microvascular damage and ischemia but also intraretinal inflammation and neuronal degeneration. Despite the increasing knowledge on the pathogenic pathways involved in DR, currently approved treatments are focused only on its late-stage vasculopathic complications, and a single molecular target, vascular endothelial growth factor (VEGF), has been extensively studied, leading to drug development and approval. In this review, we discuss the state of the art of research on neuroinflammation and neurodegeneration in diabetes, with a focus on pathophysiological studies on human subjects, *in vivo* imaging biomarkers, and clinical trials on novel therapeutic options.

## Introduction

Diabetic retinopathy (DR) is the most common complication of diabetes mellitus (DM) and the leading cause of preventable blindness in developed countries. In the past, DR was regarded as a microangiopathy because its clinically detectable lesions are mainly vascular: even nowadays, its diagnosis and staging are still based on vascular abnormalities observed by fundoscopy. However, the American Diabetes Association has recently defined DR as a highly tissue-specific neurovascular complication of DM involving progressive disruption of the interdependence between multiple cell types in the retina (Solomon et al., 2017). Indeed, the retinal neurovascular unit (NVU) is composed of neurons, glial cells, and the intraretinal vascular network (Hawkins and Davis, 2005; Antonetti et al., 2012). Recent evidence suggests that DR is the result of a global dysfunction of the NVU: the activation of glial cells (astrocytes, Müller cells, and microglia) and degeneration of neural elements (ganglion, bipolar, horizontal, and amacrine cells) are distinct pathogenic events (and therefore therapeutic targets) that interplay with microvascular phenomena (Gardner and Davila, 2017; Nian et al., 2021), leading to the development of diabetic retinal disease (DRD).

Current treatments for DR are available only for its vision-threatening complications, such as diabetic macular edema (DME) and proliferative diabetic retinopathy (PDR). A better understanding of pathologic retinal changes in diabetes is crucial for the identification of novel pharmacological targets in order to prevent the development of late-stage complications, improve functional and anatomical outcomes and maybe slow the progression of irreversible retinal neurodegeneration. In this review, we discuss the state of the art of research on the role of neuroinflammation and neurodegeneration in DR, with a focus on pathophysiological studies on human subjects, *in vivo* imaging biomarkers, and clinical trials on novel therapeutic options.

## Methods

We searched all English language and human subject scientific literature on PubMed library using the following keywords: DRD, DR, DME, inflammation, neurodegeneration, glia, multimodal imaging, biomarker. Additional articles were identified through the references of publications selected in the first instance. The website https://clinicaltrials.gov/ was used to find all clinical trials on DR and DME targeting neuroinflammation and neuroprotection.

## Neuroinflammation in diabetic retinopathy

### Pathophysiology of retinal inflammation in diabetic retinopathy

Inflammation is a complex biological response of tissues and cells to pathogens and damaged cells that involves leucocytes, blood vessels, and many molecular mediators. Acute inflammation typically has beneficial effects in the acute setting, while becomes detrimental if persisting chronically. Initial suggestions for possible involvement of inflammation in DR came from reports of a lower incidence of DR in arthritic patients taking salicylates (Powell and Field, 1964). Experimental evidence for the presence of chronic-low grade inflammation from the early moments of DR pathogenesis began accumulating since Schröder et al. (1991) demonstrated in a murine model that leukostasis, a phenomenon that may cause retinal microvasculature dropout by monocytes and granulocytes, is present even in NPDR. Biochemical, molecular, and cellular mechanisms of neuroinflammation in DR significantly contributed to current knowledge of DR pathogenesis, are better studied in animal models, and are extensively reviewed elsewhere (Tang and Kern, 2011; Forrester et al., 2020; Pan et al., 2021). Before delving into the relationship between neuroinflammation and DR through the analysis of current evidence on clinical research in human subjects, a short overview is provided.

Neuroretinal inflammation is mediated by the retinal glia, which senses stress signals in the neural tissue (such as high glucose levels or glycated compounds, oxidative stress, and damaged cells) and secretes pro-inflammatory mediators. Retinal glia is composed of three cytotypes: astrocytes, Müller cells, and microglia. Astrocytes are not strictly of neuroepithelial origin but enter the developing retina from the optic nerve and present several fibrous processes radiating from the cell body that cover blood vessels in the superficial capillary plexus, contributing to inner retinal blood barrier (iBRB) formation (Stone and Dreher, 1987). Microglial cells are not of neuroglial origin, despite their name, but enter the retina with the mesenchymal precursors of retinal blood vessels and are believed to represent innate immune cells of the neural tissue, with a macrophage-like function (Rathnasamy et al., 2019). Müller cells are sustentacular cells that span from the external to the internal limiting membrane, connecting neurons, and vascular cells (Vecino et al., 2016).

Müller cells gliosis (activation) is a hallmark of DR, as demonstrated by a significant increase in aqueous biomarkers of glial activation (glial fibrillary acidic protein, aquaporin 1, and aquaporin 4) in aqueous humor (Vujosevic et al., 2015). Chronic hyperglycemia could induce gliosis through the formation of advanced glycation end products (AGEs) (Sorrentino et al., 2016; Xu et al., 2018), which are macromolecules that become abnormally glycated after exposure to chronically elevated blood glucose concentrations. The factors associated with AGEs formation include normal aging, degree of hyperglycemia, and glycated protein turnover (Sharma et al., 2012). Accumulated AGEs, both within retinal walls and serum, are capable of inducing pro-inflammatory responses by receptor (RAGE) dependent or independent pathways: AGEs can induce the upregulation of adhesion molecules (such as ICAM-1) on endothelial cells and directly activate leukocytes (Moore et al., 2003), while the activation of RAGE results in glial activation and inflammatory cytokines secretion. The formation of AGEs is also influenced by oxidizing conditions and reactive oxygen species (ROS) formation concentrations, thereby creating a positive feedback loop between retinal neuroinflammation and AGEs accumulation (Baynes and Thorpe, 2000).

Gliosis of Müller cells results in the secretion of pro-inflammatory cytokines, chemokines, and vascular endothelial growth factor (VEGF) (Bringmann and Wiedemann, 2012). While elevated VEGF levels directly cause iBRB instability and neovascularization development (Ozaki et al., 1997; Qaum et al., 2001), chemokines and cytokines attract and activate leukocytes. The adhesion of leukocytes to the endothelium for subsequent diapedesis is mediated by leukocyte integrins and endothelial cell adhesion molecules. Indeed, an elevated concentration of E-selectin in the plasma of diabetic subjects may play a role in the development of DR (Kasza et al., 2017). At this point, leucocytes recruited in the retinal capillaries appear to activate the Fas (CD95)/Fas-ligand pathway, eventually leading to endothelial cell apoptosis and further iBRB impairment (Joussen et al., 2003; Vincent and Mohr, 2007).

### Vitreous and aqueous molecular biomarkers of neuroinflammation in diabetic retinopathy

Several studies tried to determine the inflammatory profile of DR and DME *in vivo* by measuring cytokine concentrations on intraocular fluids (Sivalingam et al., 1990; Yuuki et al., 2001; Funatsu et al., 2002, 2009; Suzuki et al., 2011; Jonas et al., 2012; Lee et al., 2012; Schoenberger et al., 2012; Bromberg-White et al., 2013; Mao and Yan, 2014; Noma et al., 2014, 2017; Dong et al., 2015; Kim et al., 2015; Takeuchi et al., 2015; Ghodasra et al., 2016; Vujosevic et al., 2016b; Boss et al., 2017; Wu et al., 2017; Song et al., 2020). Table 1 lists all the inflammatory mediators investigated on intraocular fluids of human subjects with DR and DME. Among the many identified, the most promising is IL-6, according to multiple independent studies. Indeed, IL-6 and IL-8 are increased in the vitreous of eyes with PDR (Yuuki et al., 2001) and have an independent influence on macular volume and DME severity (Dong et al., 2015; Kim et al., 2015). However, conflicting data on the role of IL-8 can be found in literature (Ghodasra et al., 2016; Vujosevic et al., 2016b). Other studies suggest that glial activation biomarkers (MIP-1β, GM-CSF, RANTES, and sTNF-RII) in the aqueous humor of patients with DR (Vujosevic et al., 2016b), which are presumably secreted under the inflammatory drive of IL-6 and IL-8 from Müller cells (Boss et al., 2017), could be associated with the concentration of neurotrophic mediators (such as Nerve Growth Factor, Brain-Derived Neurotrophic Factor, and Glial cell–Derived Neurotrophic Factor).

**TABLE 1:** Summary of inflammation-related cytokines, chemokines, and growth factors investigated in the aqueous humor (A) or vitreous (V) of human patients with non-proliferative diabetic retinopathy (NPDR), proliferative diabetic retinopathy (PDR), and diabetic macular edema (DME).

Cytokine, chemokine, growth factor	Diabetic retinopathy stage						References
	NPDR		PDR		DME		
	A	V	A	V	A	V	
*ADAM11*							Ghodasra et al., 2016
*CX3CL1 (Fractalkine)*							Ghodasra et al., 2016
*CXCL1 (GRO)*							Schoenberger et al., 2012; Bromberg-White et al., 2013; Ghodasra et al., 2016
*CXCL10 (IP-10)*							Suzuki et al., 2011; Jonas et al., 2012; Schoenberger et al., 2012; Bromberg-White et al., 2013; Dong et al., 2015; Kim et al., 2015; Ghodasra et al., 2016; Vujosevic et al., 2016b; Noma et al., 2017
*CXCL12 (SDF-1)*							Jonas et al., 2012
*EGF*							Jonas et al., 2012; Lee et al., 2012; Ghodasra et al., 2016
*Eotaxin (CCL11)*							Suzuki et al., 2011; Schoenberger et al., 2012; Bromberg-White et al., 2013; Dong et al., 2015; Ghodasra et al., 2016
*FGF2 (bFGF, FGF-*β)							Sivalingam et al., 1990; Suzuki et al., 2011; Jonas et al., 2012; Lee et al., 2012; Bromberg-White et al., 2013; Dong et al., 2015; Ghodasra et al., 2016
*FLT3L*							Bromberg-White et al., 2013; Ghodasra et al., 2016
*G-CSF*							Suzuki et al., 2011; Dong et al., 2015; Ghodasra et al., 2016
*GM-CSF*							Suzuki et al., 2011; Bromberg-White et al., 2013; Dong et al., 2015; Ghodasra et al., 2016; Vujosevic et al., 2016b
*HGF*							Jonas et al., 2012
*ICAM-1 (CD54)*							Funatsu et al., 2009; Jonas et al., 2012; Noma et al., 2014; Noma et al., 2017; Song et al., 2020
*IFN-*α							Jonas et al., 2012; Lee et al., 2012; Bromberg-White et al., 2013; Ghodasra et al., 2016
*IFN-*β							Jonas et al., 2012
*IFN-*γ							Suzuki et al., 2011; Jonas et al., 2012; Lee et al., 2012; Dong et al., 2015; Takeuchi et al., 2015; Ghodasra et al., 2016; Vujosevic et al., 2016b; Wu et al., 2017
*IL-1*α							Jonas et al., 2012; Bromberg-White et al., 2013; Ghodasra et al., 2016; Vujosevic et al., 2016b
*IL-1*β							Suzuki et al., 2011; Jonas et al., 2012; Mao and Yan, 2014; Dong et al., 2015; Takeuchi et al., 2015; Ghodasra et al., 2016; Vujosevic et al., 2016b; Boss et al., 2017; Wu et al., 2017
*IL-1RA*							Suzuki et al., 2011; Dong et al., 2015; Ghodasra et al., 2016
*IL-2*							Suzuki et al., 2011; Jonas et al., 2012; Lee et al., 2012; Dong et al., 2015; Ghodasra et al., 2016; Wu et al., 2017
*IL-3*							Jonas et al., 2012; Ghodasra et al., 2016; Vujosevic et al., 2016b
*IL-4*							Suzuki et al., 2011; Jonas et al., 2012; Takeuchi et al., 2015; Dong et al., 2015
*IL-5*							Suzuki et al., 2011; Jonas et al., 2012; Lee et al., 2012; Dong et al., 2015; Ghodasra et al., 2016; Wu et al., 2017
*IL-6*							Yuuki et al., 2001; Funatsu et al., 2002; Funatsu et al., 2005; Funatsu et al., 2009; Suzuki et al., 2011; Jonas et al., 2012; Lee et al., 2012; Schoenberger et al., 2012; Bromberg-White et al., 2013; Dong et al., 2015; Kim et al., 2015; Takeuchi et al., 2015; Ghodasra et al., 2016; Vujosevic et al., 2016b; Boss et al., 2017; Noma et al., 2017; Wu et al., 2017; Song et al., 2020
*IL-6RA*							Vujosevic et al., 2016b
*IL-7*							Suzuki et al., 2011; Bromberg-White et al., 2013; Dong et al., 2015; Ghodasra et al., 2016
*IL-8*							Yuuki et al., 2001; Suzuki et al., 2011; Jonas et al., 2012; Lee et al., 2012; Schoenberger et al., 2012; Bromberg-White et al., 2013; Dong et al., 2015; Kim et al., 2015; Ghodasra et al., 2016; Vujosevic et al., 2016b; Boss et al., 2017; Noma et al., 2017; Song et al., 2020
*IL-9*							Suzuki et al., 2011; Dong et al., 2015; Ghodasra et al., 2016
*IL-10*							Suzuki et al., 2011; Jonas et al., 2012; Bromberg-White et al., 2013; Mao and Yan, 2014; Dong et al., 2015; Takeuchi et al., 2015; Ghodasra et al., 2016; Vujosevic et al., 2016b; Wu et al., 2017; Song et al., 2020
*IL-12p40*							Suzuki et al., 2011; Jonas et al., 2012; Bromberg-White et al., 2013; Dong et al., 2015; Ghodasra et al., 2016; Vujosevic et al., 2016b
*IL-12p70*							Suzuki et al., 2011; Jonas et al., 2012; Lee et al., 2012; Dong et al., 2015; Vujosevic et al., 2016b; Noma et al., 2017
*IL-13*							Suzuki et al., 2011; Lee et al., 2012; Dong et al., 2015; Ghodasra et al., 2016; Noma et al., 2017
*IL-15*							Suzuki et al., 2011; Dong et al., 2015; Ghodasra et al., 2016; Vujosevic et al., 2016b
*IL-17A*							Suzuki et al., 2011; Dong et al., 2015; Takeuchi et al., 2015; Ghodasra et al., 2016
*IL-21*							Takeuchi et al., 2015
*IL-22*							Takeuchi et al., 2015
*IL-23*							Takeuchi et al., 2015
*IL-24*							Takeuchi et al., 2015
*IL-31*							Takeuchi et al., 2015
*IL-33*							Takeuchi et al., 2015
*M-CSF*							Vujosevic et al., 2016b
*MCP-1 (CCL2)*							Funatsu et al., 2009; Suzuki et al., 2011; Jonas et al., 2012; Lee et al., 2012; Schoenberger et al., 2012; Bromberg-White et al., 2013; Noma et al., 2014; Dong et al., 2015; Kim et al., 2015; Vujosevic et al., 2016b; Noma et al., 2017; Song et al., 2020
*MCP-2 (CCL8)*							Vujosevic et al., 2016b
*MCP-3 (CCL7)*							Jonas et al., 2012; Bromberg-White et al., 2013; Ghodasra et al., 2016; Vujosevic et al., 2016b
*MDC (CCL22)*							Bromberg-White et al., 2013
*MIF*							Jonas et al., 2012
*MIG*							Jonas et al., 2012
*MIP-1*α *(CCL3)*							Suzuki et al., 2011; Lee et al., 2012; Dong et al., 2015; Ghodasra et al., 2016; Vujosevic et al., 2016b
*MIP-1*β *(CCL4)*							Suzuki et al., 2011; Bromberg-White et al., 2013; Dong et al., 2015; Ghodasra et al., 2016; Vujosevic et al., 2016b
*MIP-1*δ *(CCL15)*							Vujosevic et al., 2016b
*MMP-1*							Jonas et al., 2012
*MMP-9*							Jonas et al., 2012
*PAI-1*							Jonas et al., 2012
*PDGF-AA*							Schoenberger et al., 2012; Lee et al., 2012; Kim et al., 2015; Ghodasra et al., 2016; Noma et al., 2017
*PDGF-BB*							Suzuki et al., 2011; Jonas et al., 2012; Dong et al., 2015; Ghodasra et al., 2016
*PGE*_2_							Schoenberger et al., 2012
*PlGF*							Jonas et al., 2012; Noma et al., 2017
*PTX3*							Noma et al., 2014
*RANTES (CCL5)*							Suzuki et al., 2011; Dong et al., 2015; Ghodasra et al., 2016; Vujosevic et al., 2016b
*TGF-*α							Jonas et al., 2012; Lee et al., 2012; Ghodasra et al., 2016
*TGF-*β*1*							Jonas et al., 2012; Vujosevic et al., 2016b; Song et al., 2020
*TNF-*α							Yuuki et al., 2001; Suzuki et al., 2011; Schoenberger et al., 2012; Dong et al., 2015; Takeuchi et al., 2015; Ghodasra et al., 2016; Vujosevic et al., 2016b; Boss et al., 2017; Wu et al., 2017
*TNF-*β							Ghodasra et al., 2016
*sCD40L*							Bromberg-White et al., 2013; Takeuchi et al., 2015
*sTNF-RI*							Vujosevic et al., 2016b
*sTNF-RII*							Vujosevic et al., 2016b
*TRAIL*							Jonas et al., 2012
*VCAM-1*							Jonas et al., 2012; Song et al., 2020

Green: increased in DR; red: not significantly increased, decreased or undetectable; yellow: conflicting data between studies; gray: no data.

The other promising molecule that appears to have a role in the development of DME is ICAM-1, a ligand for LFA-1 integrin receptor that mediates leukocytes’ adhesion to endothelial cells. Its concentration in the vitreous fluid is significantly higher in eyes with DME than in diabetic eyes without retinopathy and is correlated with the degree of fluorescein leakage and macular thickness (Funatsu et al., 2009). Also, Jonas and associates found that ICAM-1 concentration in the aqueous humor was the most associated with macular thickness (Jonas et al., 2012).

*In vivo* studies on cytokines in intraocular fluids paved the way for the identification of an enormous number of novel molecular targets. However, few anti-inflammatory targeted therapies have been developed by pharmaceutical companies, even fewer are in late-phase clinical trials, and only old molecules acting more broadly on the inflammatory cascade, such as corticosteroids, have been approved for intravitreal use (see also “anti-inflammatory compounds” section in “novel treatment strategies” chapter). The reason could be linked to the lack of robust studies with adequate samples on cytokine levels in eyes at different stages of DR, leading to contradictory pieces of information due to the enormous number of small studies on the topic (Table 1). Moreover, many of these research indifferently sampled cytokines from the aqueous humor rather than the vitreous. Despite one study suggested that aqueous humor concentration of some inflammatory cytokines may be correlated with that in the vitreous chamber (Funatsu et al., 2005), subsequent works demonstrated the fallacy of assuming that the concentration of proteins in the aqueous correlate with their counterparts in the vitreous (Noma et al., 2010; Ecker et al., 2011). Therefore, caution should be taken when comparing studies on cytokine levels in different intraocular fluids and further research in this area should investigate which cytokine, in which compartment may be used as a biomarker of retinal inflammation in DR.

In conclusion, retinal inflammation can be considered a key pathogenic factor in DR and especially in the progression to PDR and development of DME. Further pharmacological research is needed to bring novel therapeutic options into clinical practice.

### Multimodal imaging biomarkers of neuroinflammation in diabetic retinopathy

There is a growing scientific interest in the possibility of identifying retinal inflammation *in vivo*, by means of multimodal imaging, which includes optical coherence tomography (OCT), OCT-angiography (OCTA), fluorescein angiography (FA), indocyanine-green angiography (ICGA), and confocal MultiColor imaging among the others.

Retinal hyperreflective foci (HF) are small (<30 μm), punctiform lesions with reflectivity similar to that of NFL, scattered throughout the neuroretina, and visible on OCT B-scans. HF can be distinguished from hard intraretinal exudates and microaneurysms and have been proposed to represent aggregates of microglial cells (Vujosevic et al., 2017a; Pilotto et al., 2020). In diabetic eyes without clinical retinopathy, HF can be identified in the inner retina, where most microglial cells are present, while DR is associated with their outer retinal migration (Vujosevic et al., 2013). HF number increases with DR and DME severity (Schreur et al., 2020).

The inflammatory origin of HF is debated but supported by a histopathologic study that investigated microglial activation in human DR and found an increased number of hypertrophic microglial cells scattered in the inner retinal layers, which were also present in outer retinal layers in later stages of the disease (Zeng et al., 2008). However, it must be noted that immunolabeled microglial cells in retinal sections did not clearly resemble HF and were often located around retinal vasculature, microaneurysms, intraretinal hemorrhages, cotton-wool spots, and neovascularizations, which have not been described for HF, although no OCT-based investigation specifically addressed this area of uncertainty (Zeng et al., 2008). Another research supporting this hypothesis reported an association between aqueous humor concentration of soluble CD14, a molecule involved in the cellular recognition of inflammatory signals (such as lipopolysaccharide), and HF number in diabetics with DME (Lee et al., 2018).

Other works specifically investigated the functional, prognostic and predictive role of HF. In DME, HF number shows an inverse correlation with macular sensitivity, possibly linking microglial inflammatory response to functional neuroretinal impairment (Vujosevic et al., 2016a), but predicts a higher increase in sensitivity thresholds after intravitreal dexamethasone treatment (Vujosevic et al., 2017c). Also, the presence of numerous HF at baseline predicts a worse visual acuity at the end of follow-up in DME treated with observation (Busch et al., 2020). However, a recent systematic review found that it is still unclear whether HF presence in DME can predict treatment outcomes, even though their number decreases after treatment (Huang et al., 2022).

Vitreous HF are another potential biomarker of neuroinflammation. The resolution of spectral-domain OCT is in the range of 5–7 μm and, thus, has the potential of imaging leukocytes which can be up to 10–30 μm in size. These leukocytes would appear as vitreous HF, larger and brighter than the usual background speckle and about 20 μm in diameter (Saito et al., 2013). A study by Mizukami et al. (2017) found a correlation between the average number of vitreous HF and the severity of DR. Similarly, macrophage-like cells (MLCs) at the vitreoretinal interface can be seen using en-face OCT. Their signal corresponds to a ramified morphology and a recent study found that MLC density is higher in PDR compared with controls, diabetics without retinopathy, and NPDR (Ong et al., 2021). Interestingly, MLCs are more likely to localize on blood vessels and in perivascular areas than in ischemic areas (Ong et al., 2021).

Also, the OCT pattern of DME may become a key feature to guide treatment and predict outcomes. Current treatments for DME include intravitreal therapies that target different aspects of its pathophysiology: anti-VEGF for vasogenic edema and corticosteroids for inflammatory edema (Romero-Aroca et al., 2016; Schmidt-Erfurth et al., 2017). DME can present with various patterns on OCT, including subretinal fluid (SRF) accumulation, which is visible on OCT as a hyporeflective area under the neuroretina (Otani et al., 1999). This pattern is associated with higher concentrations of CXCL10, IL-6, IL-8, and PDGF-AA but not VEGF (Sonoda et al., 2014; Kim et al., 2015) and by a higher number of HF (Vujosevic et al., 2017b), suggesting that inflammation plays a pivotal role in the development of at least some cases of DME. Indeed, dexamethasone treatment of SRF-associated DME is associated with a greater improvement of CMT (central macular thickness), retinal HF, and disorganization of inner retinal layers and cysts area with respect to ranibizumab treatment (Vujosevic et al., 2020).

Choroidal OCT biomarkers of inflammation have been proposed to monitor the response of DME to intravitreal corticosteroids: choroidal HF, which again are thought to represent inflammatory aggregates (Lee et al., 2018; Schreur et al., 2018), and choroidal vascularity index (CVI), measured as the ratio of choroid occupied by vessels and providing information on choroidal congestion (Kim et al., 2018; Iovino et al., 2020). A recent prospective study from our research group evaluated the relationship between OCT biomarkers of inflammation in DME and the response to treatment with fluocinolone acetonide (FAc) 0.19 mg intravitreal implant over 1 year of follow-up. Good responders tend to show higher choroidal HF and lower CVI than poor responders and both metrics do not change over the follow-up in poor responders to FAc implant (Arrigo et al., 2020). A subsequent investigation on non-naïve DME eyes treated with anti-VEGF and/or dexamethasone or FAc implant found that foveal eversion is a negative biomarker associated with a higher prevalence of persistent DME (Arrigo et al., 2021). Since DME with foveal eversion is associated with a cytokine profile similar to that observed in inflammatory diseases such as uveitis or Irvine-Gass syndrome (Kiire et al., 2014), it could represent a sign of Müller cells impairment due to a chronic inflammatory *milieu* in the retinal tissue (Arrigo et al., 2021). These facts underline the compelling need for a better understanding of DME physiopathology and for validated OCT biomarkers that would guide the therapeutic strategy in accordance with the chorioretinal inflammatory profile.

Recently, it has been proposed that macular perfusion in DME eyes increases after intravitreal FAc administration, owing to a reduced leukostasis (Brambati et al., 2022). DME eyes have a decreased vessel density (VD), mainly at the deep capillary plexus (DCP) (Lee et al., 2016; AttaAllah et al., 2019), but OCTA has limited reliability in DME because cystic cavities interfere with flow detection from deeper retinal layers and with correct anatomical segmentation of retinal layers and vascular plexuses (Spaide, 2015; de Carlo et al., 2016; Tey et al., 2019). Previous studies reported conflicting data on VD change after intravitreal treatment for DME (Toto et al., 2017; Mastropasqua et al., 2018). However, Vujosevic et al. (2020) performed all quantitative OCTA analyses after image compensation for artifacts determined by intraretinal cysts and found a decrease in VD at DCP after dexamethasone but not ranibizumab treatment. The authors excluded an ischemic effect of dexamethasone because the VD decrease was not accompanied by a difference in vessel length or caliber between the two treatments (Vujosevic et al., 2020). Therefore, further studies on larger cohorts and novel methodological approaches are needed to clarify the relationship between macular perfusion and anti-inflammatory treatments for DME.

## Neurodegeneration in diabetic retinopathy

**FIGURE 1:**
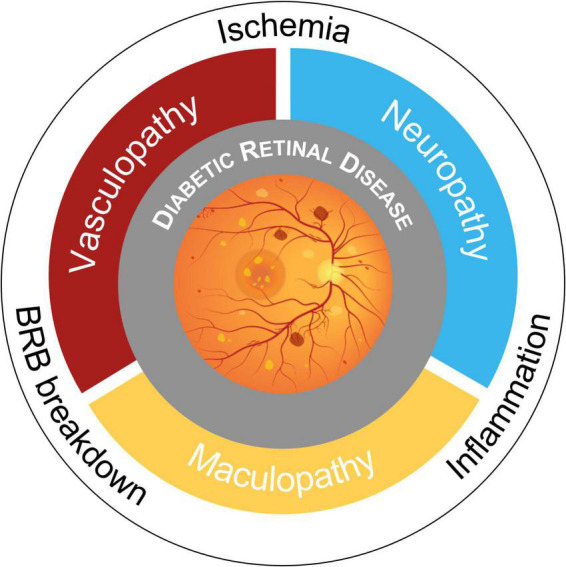
Schematic overview of pathogenic mechanisms and phenotypes in Diabetic Retinal Disease (DRD). Retinal complications of diabetes have historically been considered microangiopathic in nature. Now, the paradigm is shifting toward a novel view of DRD as a tissue-specific neurovascular complication due to blood-retinal barrier (BRB) breakdown, retinal and optic nerve head ischemia, and neuroinflammation.

Retinal neurodegeneration is a process that features reactive gliosis, diminished neuronal function, and neuronal loss and it has long been known that in the diabetic retina the development of microangiopathic findings is preceded by neuronal functional changes and apoptosis (Simonsen, 1980; Barber et al., 1998; Carrasco et al., 2007). Intrinsic retinal microvasculature lacks autonomic innervation and vessel dilation is guaranteed by glia-mediated autoregulatory signals from neurons in case of high metabolic stress (functional hyperemia) (Metea and Newman, 2007; Newman, 2013). A number of studies demonstrated that this mechanism of neurovascular coupling is impaired even in the early stages of DR (Bek, 2017) and the term “DRD” has been proposed to express all neurovascular pathologic changes in diabetes (Figure 1; Abramoff et al., 2018; Levine et al., 2022). However, photographic imaging of clinically evident vascular lesions has limited the study and understanding of diabetic retinal neuropathy.

Direct evidence for photoreceptor dysfunction early in the course of DRD was demonstrated in the 80′, with two studies showing a decreased contrast and color sensitivity in diabetics without retinopathy (Roy et al., 1986; Trick et al., 1988). Subsequently, photoreceptors’ transduction abnormalities in patients with early DR were detected by means of full-field electroretinogram (ERG) (Holopigian et al., 1997). Recently, multifocal ERG confirmed the presence of functional alterations even from the preclinical stage of DR, though in presence of an altered superficial capillary plexus (Reis et al., 2014). However, it must be noted that a significant proportion of diabetic patients with early microvascular disease have no detectable neuronal dysfunction on multifocal ERG (Santos et al., 2017).

On the other hand, an accurate *in vivo* assessment of the inner retinal status in diabetic eyes became possible only with the advent of OCT, despite diabetes-associated damage to nerve fiber layer (NFL) had already been demonstrated (Özdek et al., 2002; Takahashi et al., 2006). Macular NFL and ganglion cell/inner plexiform layer (GCIPL) thickness are decreased in diabetic eyes, even in absence of vascular signs (van Dijk et al., 2009; Vujosevic and Midena, 2013; Chhablani et al., 2015; Picconi et al., 2017; Scarinci et al., 2017). Sohn et al. (2016) found that this form of retinal diabetic neuropathy is progressive: diabetics without retinopathy display a loss of 0.25 μm/year in macular NFL and 0.29 μm/year in GCIPL over 4 years of follow-up. These neuroretinal changes may be related to poor metabolic control (Lonneville et al., 2003; Picconi et al., 2017).

However, the relationship between diabetic neuropathy and vasculopathy is still debated: neurodegeneration could be due to subclinical changes in intraretinal microvasculature that can be detected and quantified with the aid of OCTA. Indeed, recent work by Marques et al. (2022) demonstrated that the progression of neuroretinal thinning in patients with NPDR is associated with a decrease in VD at the DCP. Several investigations tried to clarify the pathogenetic sequence by studying the optic nerve head in diabetics, because of the intimate relationship between neural and vascular elements in the peripapillary area. Two investigations on DR (Rodrigues et al., 2019; Frizziero et al., 2021) found a reduction of both radial peripapillary capillary plexus (RPC) vascular density and peripapillary NFL thickness, but only one (Rodrigues et al., 2019) was able to demonstrate a direct correlation between the two parameters. Similarly, two studies on optic nerve head neurovascular tissue in diabetics without retinopathy were published in 2019: one reported a reduction of peripapillary VD in absence of a NFL loss (Li et al., 2019), confirming previous results (Takahashi and Chihara, 2008; Vujosevic and Midena, 2013), while the other suggested that both peripapillary VD and NFL thickness are decreased with respect to controls, albeit the former could be more prominent than the latter (Cao et al., 2019).

Therefore, to present knowledge, retinal neurodegeneration can only be considered as a different pathologic feature of DRD, at least partly independent from microvascular alterations. This new view of DR underpins the need for better phenotyping and stratification of DRD in order to identify the subset of patients with chronic neurodysfunction and validate biomarkers to measure therapy outcomes, considering the emergent development of neuroprotective drugs.

## Novel treatment strategies

Currently, a robust amount of research is conducted on developing new pharmacological agents for DR and DME. At the moment the only strategy to prevent or halt the progression of DR is intensive blood glucose control (Diabetes Control and Complications Trial Research Group, 1995) while effective therapies are available to treat only late-stage complications related to ischemia and DME, even though the incidence of persistent DME after 1 or 2 years of anti-VEGF treatment is still very high (Dugel et al., 2016; Bressler et al., 2018). Several alternative strategies have been tested in the pre-clinical setting and here we summarize the advancement in research on novel therapeutics targeting inflammation (Table 2) and neurodegeneration (Table 3) in DR and DME, with a focus on those under investigation in clinical trials.

**TABLE 2:** Clinical trials on novel drugs targeting inflammation in diabetic retinopathy.

Drug (administration route)	Category or mechanism	Title	Phase	Identifier (status)
*ASP8232 (PO)*	Vascular adhesion protein-1 inhibitor	A study to evaluate ASP8232 in reducing central retinal thickness in subjects with diabetic macular edema (DME) (VIDI)	II	NCT02302079 (completed)
*AXT107 (IVT)*	Integrin receptor antagonist	Safety and bioactivity of AXT107 in subjects with diabetic macular edema (CONGO)	I-IIa	NCT04697758 (active)
*Dexamethasone (IVT implant)*	Steroidal anti-inflammatory	Three-Year, randomized, sham-controlled trial of dexamethasone intravitreal implant in patients with diabetic macular edema	III	NCT00168337, NCT00168389 (completed)
*EBI-031 (IVT)*	IL-6 inhibitor	Safety study of intravitreal EBI-031 given as a single or repeat injection to subjects with diabetic macular edema	I	NCT02842541 (withdrawn)
*Fluocinolone acetonide (IVT implant)*	Steroidal anti-inflammatory	Fluocinolone acetonide implant compared to sham injection in patients with diabetic macular edema (FAME)	III	NCT00344968 (completed)
*Infliximab (IV)*	Anti-TNF-α	Treatment of refractory diabetic macular edema with infliximab	III	NCT00505947 (completed)
*KVD001 (IVT)*	Plasma kallikrein inhibitor	Study of the intravitreal plasma kallikrein inhibitor, KVD001, in subjects with center-involving diabetic macular edema (ciDME)	II	NCT03466099 (completed)
*Nepafenac (T)*	COX-inhibitor	A phase II evaluation of topical non-steroidal anti-inflammatories in eyes with non-central involved diabetic macular edema	II	NCT01331005 (completed)
*PF-04634817 (PO)*	Chemokine receptors antagonist	A phase 2, multi-center study to compare the efficacy and safety of a chemokine CCR2/5 receptor antagonist with ranibizumab in adults with diabetic macular edema	II	NCT01994291 (completed)
*Risuteganib (IVT)*	Integrin receptor antagonist	Phase 2 randomized clinical trial of luminate as compared to avastin in the treatment of diabetic macular edema	IIb	NCT02348918 (completed)
*SF0166 (T)*	Integrin receptor antagonist	Safety and exploratory efficacy study of SF0166 for the treatment of diabetic macular edema (DME)	I-II	NCT02914613 (completed)
*THR-149 (IVT)*	Plasma kallikrein inhibitor	A study to evaluate the safety of THR-149 in subjects with diabetic macular edema	I	NCT03511898 (completed)
*THR-687 (IVT)*	Integrin receptor antagonist	A phase 1, open-label, multicenter, dose escalation study to evaluate the safety of a single intravitreal injection of THR-687 for the treatment of diabetic macular edema	I	NCT03666923 (completed)
*Tocilizumab (IV)*	IL-6 receptor antagonist	Ranibizumab for edema of the macula in diabetes: Protocol 4 with tocilizumab: The READ-4 study (READ-4)	II	NCT02511067 (withdrawn)
*Triamcinolone acetonide (IVT)*	Steroidal anti-inflammatory	Intravitreal triamcinolone acetonide vs. laser for diabetic macular edema	III	NCT00367133 (completed)

IV, intravenous; IVT, intravitreal; PO, per os; T, topical.

**TABLE 3:** Clinical trials on novel drugs targeting neurodegeneration in diabetic retinopathy.

Drug (administration route)	Category or mechanism	Title	Phase	Identifier (status)
*Brimonidine (topical)*	α_2_-adrenergic agonist	Trial to assess the efficacy of neuroprotective drugs administered topically to prevent or arrest diabetic retinopathy (EUROCONDOR)	II-III	NCT01726075 (completed)
*Cibinetide (subcutaneous)*	Erythropoietin-derived peptide	A phase II clinical trial on the use of ARA 290 for the treatment of diabetic macular edema (ARA 290-DMO)	II	EudraCT 2015-001940-12 (completed)
*Citicoline and vitamin B_12_ (topical)*	Phosphatidylcholine precursor	Long-term retinal changes after topical citicoline administration in patients with mild signs of diabetic retinopathy in type 1 diabetes mellitus	Not applicable	NCT04009980 (completed)
*Elamipretide (topical)*	Mitochondrial cardiolipin stabilizer	A study of MTP-131 topical ophthalmic solution in subjects with diabetic macular edema and non-exudative intermediate age-related macular degeneration (SPIOC-101)	I-II	NCT02842541 (completed)
*Somatostatin (topical)*	Endogenous neuroprotective hormone	Trial to assess the efficacy of neuroprotective drugs administered topically to prevent or arrest diabetic retinopathy (EUROCONDOR)	II-III	NCT01726075 (completed)

### Anti-inflammatory compounds

#### Corticosteroids

Despite an early clinical trial (NCT00367133) on intravitreal triamcinolone demonstrated its inferiority to focal/grid laser photocoagulation (Diabetic Retinopathy Clinical Research Network, 2008), intravitreal corticosteroids are the only category of pure anti-inflammatory drugs to be approved for clinical use in DR complicated by DME: FAc 0.2 mg implant (Iluvien; NCT00367133) (Campochiaro et al., 2011) and dexamethasone 0.7 mg (Ozurdex; NCT00168337, NCT00168389) (Boyer et al., 2014). Their ability to reduce intraocular inflammation has been extensively demonstrated. A study on diabetic rats demonstrated that dexamethasone reduces retinal leukostasis and vascular permeability (Wang et al., 2008), while *in vivo* effects of intravitreal corticosteroids in human subjects have been investigated by measuring the concentration of proinflammatory cytokines in intraocular fluids of eyes with DME. Indeed, triamcinolone decreases the aqueous levels of IL-6 and MCP-1 (Sohn et al., 2011), and FAc those of IL-6, IP-10, MCP-1, and ICAM-1 in the vitreous (Deuchler et al., 2022). Since the pathogenesis of DME is not only vasogenic but also inflammatory and around 40% of patients treated show suboptimal visual acuity improvements when treated with anti-VEGF (Gonzalez et al., 2016), corticosteroids use has become increasingly important, especially in chronic DME (Cunha-Vaz et al., 2014) and in DME displaying biomarkers of retinal inflammation (Arrigo et al., 2020). However, 20% of eyes receiving FAc experience persistent or recurrent DME in the first year after injection and require additional anti-VEGF treatments (Cicinelli et al., 2020). Therefore, new drugs targeting other pathways of retinal inflammation are needed.

#### Non-steroidal anti-inflammatory drugs

NSAIDs mechanism of action pertains the inhibition of cyclooxygenases, which are enzymes involved in the synthesis of prostaglandins, one of the key biological mediators of inflammation. Prostaglandin E2 (PGE_2_) signaling seems to be involved in the pathogenesis of DR (Schoenberger et al., 2012; Wang et al., 2019) but clinical investigations on the role of topical NSAIDs in DME suggest that these medications have scant therapeutic potential. Indeed, topical nepafenac showed no effect on visual function in DRCR Network Protocol R (NCT01331005), just like bromfenac in a smaller pilot study (Friedman et al., 2015; Pinna et al., 2017). The poor efficacy may be due to the topical administration route, but a small, randomized trial on intravitreal diclofenac confirmed no beneficial effect on visual function, despite a reduction of DME (Elbendary and Shahin, 2011).

#### Cytokine, chemokine, and adhesion molecules inhibitors

IL-6 is a key molecule that leads to retinal inflammation in DR and DME since it has been found elevated in vitreous in several studies, as described previously (Koleva-Georgieva et al., 2011; Sonoda et al., 2014). Two registered clinical trials investigated the blockage of the IL-6 pathway with monoclonal antibodies in DME: both NCT02842541 for EBI-031 and NCT02511067 for tocilizumab are withdrawn at the present moment.

TNF-α is another inflammatory cytokine secreted by activated macrophages and monocytes. Its serum concentrations correlate with the presence and severity of DR (Koleva-Georgieva et al., 2011) and a phase III trial (NCT00505947) demonstrated that intravenous anti-TNF-α monoclonal antibody infliximab significantly improved visual acuity over placebo in patients with DME (Sfikakis et al., 2010). Interestingly, a subsequent retrospective study from Wu et al. (2011) found that intravitreal infliximab (and adalimumab) does not improve visual acuity or CMT in refractory DME.

Integrin receptors are transmembrane adhesion proteins that mediate cell-to-cell and cell-to-extracellular matrix attachment and intracellular signal transduction, playing an essential role in leukocytes’ adhesion to the endothelium and extravasation. Most of their ligands are proteins containing arginine-glycine-aspartate (RGD) or leucine-aspartate-valine (LDV) motifs (van Hove et al., 2021). Risuteganib (Luminate) is an intravitreal antagonist of RGD integrin receptors and a phase IIb trial in DME (NCT02348918) showed its non-inferiority to bevacizumab with respect to visual acuity improvement at 24 weeks of follow-up (Quiroz-Mercado et al., 2018). THR-687 is another RGD integrin receptors antagonist that demonstrated an excellent safety profile with no dose-limiting toxicities along with a rapid gain in visual acuity that lasted about 3 months in a phase I trial (NCT03666923) (Khanani et al., 2021). Also OTT-166 (formerly SF0166) is an antagonist that demonstrated biological effects after topical administration in an early clinical trial (NCT02914613) (Edwards et al., 2018), while AXT107 is currently under investigation in a phase I/IIa trial (NCT04697758).

Vascular adhesion protein-1 (VAP-1) is an endothelial and soluble enzyme with amine oxidase activity found elevated in the serum of diabetic patients and vitreous of eyes with PDR (Murata et al., 2012; Luo et al., 2013) and involved in leukocyte diapedesis and vessel leakage (Noda et al., 2009). Its oral inhibitor ASP8232 had no effect alone or in combination with ranibizumab in a phase II trial on DME (NCT02302079) (Nguyen et al., 2019).

Chemokine receptor type 2 and type 5 (CCR2 and CCR5) are expressed by monocytes and play a key role in homing inflammatory cells to retinal tissue (Rangasamy et al., 2014). Since the concentration of CCR2 and CCR5 ligands (monocyte chemoattractant protein-1 and RANTES, respectively) is elevated in the vitreous and aqueous humor of patients with DR and DME (Funatsu et al., 2009; Roh et al., 2009) and CCR2 has been implicated in VEGF production and vascular leakage (Krause et al., 2014), a phase II trial on CCR2/5 dual antagonist in DME has been performed (NCT01994291). Treatment with the CCR2/5 inhibitor was associated with a modest improvement in visual acuity, though inferior to intravitreal ranibizumab, despite the high level of CCR2 antagonism observed in the study (Gale et al., 2018).

#### Kallikrein-kinin system inhibitors

Bradykinin (BK) plays a role in vascular inflammation, ischemic vasogenic edema, and angioedema. BK is cleaved from high-molecular-weight kininogen by the proteolytically active plasma kallikrein (PKal) and stimulates two receptors, B1R and B2R: the former is constitutively expressed whereas the latter is regulated by inflammatory mediators such as IL-1β or TNF-α (Abdulaal et al., 2016). PKal concentration is increased in the vitreous of DME eyes and BK-induced retinal thickening in mice is not affected by the blockade of VEGF receptor (Kita et al., 2015). Indeed, the inhibition of B1R has been shown to inhibit retinal inflammation in the animal model (Pouliot et al., 2012) and intravitreal KVD001, which act upstream in the pathway as a PKal inhibitor, showed promising results in a phase Ib (NCT02193113) and II (NCT03466099) trial on DME (Sun et al., 2019). Also THR-149, a novel bicyclic peptide inhibitor of PKal, demonstrated clinical potential in a phase I trial (NCT03511898) (Dugel et al., 2021).

### Neuroprotective agents

No pharmacologic compound to halt neurodegeneration is approved for clinical use, yet some agents are under investigation.

#### Topical brimonidine and somatostatin

The EUROCONDOR phase II–III clinical trial (NCT01726075) investigated the neuroprotective potential of topically administered brimonidine (α_2_-adrenergic agonist) and somatostatin (endogenous neuroprotective hormone) in patients with type 2 diabetes and early stage or no DR (Simó et al., 2019). Indeed, animal studies have demonstrated that topical brimonidine enhances neuronal function and reduces apoptosis (Saylor et al., 2009); similarly, topical somatostatin prevents ERG abnormalities, glial activation, and neuronal apoptosis (Hernández et al., 2013). Results from the trial have shown that somatostatin and brimonidine do not prevent neurodysfunction in diabetics; however, in the subset of patients displaying abnormal mfERG at baseline, these two compounds arrested the progression of neural dysfunction (Simó et al., 2019).

#### Citicoline and vitamin B_12_

Citicoline is a precursor in the synthesis of phosphatidylcholine, a component of the neuronal plasma membrane. Since GC dysfunction seems an early event in DR (Parisi and Uccioli, 2001; Vujosevic and Midena, 2013; Sohn et al., 2016; Scarinci et al., 2017) and the topical administration of citicoline improves GC function (Parisi et al., 2018), a recent double-blind, randomized, placebo-controlled trial (NCT04009980) investigated the role of citicoline plus vitamin B_12_ eyedrops in mild DR and found a reduction of functional, structural and vascular progression over 36-months of follow-up (Parravano et al., 2020).

#### Elamipretide

Cardiolipin is a phospholipid of the inner mitochondrial membrane which has an active role in mitochondrial-dependent apoptosis (Birk et al., 2014). Elamipretide (formerly MTP-131) is a soluble tetrapeptide that selectively stabilizes mitochondrial cardiolipin and promotes efficient electron transfer and reversed the visual decline without improving glycemic control or reducing body weight in mouse models of diabetes (Alam et al., 2015). A phase I/II clinical trial has been carried out to determine the effects of topical ocular administration of elamipretide in DME (NCT02314299).

#### Cibinetide

Erythropoietin (EPO) is produced by RPE and can inhibit apoptosis of retinal neurons (Becerra and Amaral, 2002; Shen et al., 2010). EPO overexpression is an early event in the natural history of DR (Garciìa-Ramiìrez et al., 2008) and high levels of this growth factor can be found in the vitreous of DME eyes (Hernaìndez et al., 2006). However, the exogenous administration of EPO could exacerbate retinal neoangiogenesis and thrombosis (Chen et al., 2009, 2021). Cibinetide (formerly known as ARA290 and helix B surface peptide) is a non-erythrogenic EPO-derived peptide that retains tissue-protective properties (Brines et al., 2008). Treatment with an EPO-derived peptide in diabetic rats prevents glial dysfunction, microglial activation, and neuronal damage without altering hematocrit or exacerbating neovascularization (McVicar et al., 2011). A phase II clinical trial in DME (EudraCT 2015-001940-12) demonstrated safety but no improvements in BCVA, retinal sensitivity, or CMT (Lois et al., 2020).

#### Alpha-lipoic acid

Alpha-lipoic acid is a pro-energetic and antioxidant compound that seems to be a valuable neuroprotective option for Alzheimer’s disease (Hager et al., 2007; Fava et al., 2013). Moreover, alpha-lipoic acid treatment reduced VEGF levels and preserved ganglion cells, inner and outer layers in diabetic mouse retinas (Kan et al., 2017). A randomized trial on diabetic subjects receiving 300 mg of ALA orally once daily for 3 months demonstrated that contrast sensitivity remained stable in patients with type 1 DM and improved in those with type 2 while contrast sensitivity declined in diabetics without ALA supplementation; however, visual acuity and eye fundus image was stable in all studied subjects (Gêbka et al., 2014).

#### Carotenoids

Lutein, zeaxanthin, and meso-zeaxanthin are exogenous retinal antioxidants. The neuroprotective properties of lutein have been demonstrated in several preclinical studies (Muriach et al., 2006; Li et al., 2009). A retrospective study in which patients received a carotenoid supplement containing lutein 10 mg, zeaxanthin 2 mg, and meso-zeaxanthin 10 mg once a day observed an increase in CMT and mfERG responses after 2 years of treatment (Moschos et al., 2017).

## Conclusion

DR is characterized by extremely complex pathogenesis, in which microvascular damage holds a pivotal role. However, neuroinflammation and neuronal degeneration are common phenomena occurring in all DR stages, entangled with the effects of vascular exudation and retinal ischemia but at least partially independent, possibly explaining why some patients experience poor outcomes despite optimal treatment. The wide range of molecular targets prompts the development of new drugs, some of which are already under evaluation in clinical trials, in order to provide more bullets in the management of DR while *in vivo* multimodal imaging biomarkers promise a patient-tailored therapeutic strategy according to the vascular, inflammatory and neurodegenerative DRD phenotype.

## Author contributions

AAr and LB: study design, data collection, and manuscript draft. AAn, AS, EA, and AB: data collection and critical revision of the text. FB and MB: manuscript revision and study supervision. All authors contributed to the article and approved the submitted version.
